# PTH decreases *in vitro* human cartilage regeneration without affecting hypertrophic differentiation

**DOI:** 10.1371/journal.pone.0213483

**Published:** 2019-04-04

**Authors:** Marijn Rutgers, Frances Bach, Luciënne Vonk, Mattie van Rijen, Vanessa Akrum, Antonette van Boxtel, Wouter Dhert, Laura Creemers

**Affiliations:** 1 Department of Orthopedics, University Medical Center Utrecht, Utrecht, the Netherlands; 2 Department of Clinical Sciences of Companion Animals, Faculty of Veterinary Medicine, Utrecht University, Utrecht, the Netherlands; Università degli Studi della Campania, ITALY

## Abstract

Regenerated cartilage formed after Autologous Chondrocyte Implantation may be of suboptimal quality due to postulated hypertrophic changes. Parathyroid hormone-related peptide, containing the parathyroid hormone sequence (PTHrP 1–34), enhances cartilage growth during development and inhibits hypertrophic differentiation of mesenchymal stromal cells (MSCs) and growth plate chondrocytes. This study aims to determine the possible anabolic and/or hypertrophic effect of PTH on human articular chondrocytes. Healthy human articular cartilage-derived chondrocytes (n = 6 donors) were cultured on type II collagen-coated transwells with/without 0.1 or 1.0 μM PTH from day 0, 9, or 21 until the end of culture (day 28). Extracellular matrix production, (pre)hypertrophy and PTH signaling were assessed by RT-qPCR and/or immunohistochemistry for collagen type I, II, X, RUNX2, MMP13, PTHR1 and IHH and by determining glycosaminoglycan production and DNA content. The Bern score assessed cartilage quality by histology. Regardless of the concentration and initiation of supplementation, PTH treatment significantly decreased DNA and glycosaminoglycan content and reduced the Bern score compared with controls. Type I collagen deposition was increased, whereas PTHR1 expression and type II collagen deposition were decreased by PTH supplementation. Expression of the (pre)hypertrophic markers MMP13, RUNX2, IHH and type X collagen were not affected by PTH. In conclusion, PTH supplementation to healthy human articular chondrocytes did not affect hypertrophic differentiation, but negatively influenced cartilage quality, the tissues’ extracellular matrix and cell content. Although PTH may be an effective inhibitor of hypertrophic differentiation in MSC-based cartilage repair, care may be warranted in applying accessory PTH treatment due to its effects on articular chondrocytes.

## Introduction

Autologous chondrocyte implantation (ACI) is an effective treatment in patients with medium-sized cartilage defects [1]. Chondrocytes isolated from healthy non weight-bearing cartilage and re-transplanted after *in vitro* expansion ideally fill the void with hyaline neocartilage [2]. Variable results have, however, been found regarding the obtained cartilage quality, with fibrous or even hypertrophically differentiated tissue instead of healthy cartilage [3]. Similarly, MSC-based regeneration either as part of microfracture procedures or as exogenous cell source has been shown to result in hypertrophic differentiation [4]. A possible tool to prevent this unfavorable differentiation pathway may be the co-administration of parathyroid hormone related-peptide (PTHrP).

PTHrP plays an important role in early development and skeletogenesis [5,6], and is crucial in maintaining the chondrocytic phenotype in native cartilage. In the growth plate, a highly organized cartilage structure that enables longitudinal bone growth, PTHrP maintains chondrocytes in a proliferating state and prevents hypertrophic differentiation and bone formation [7]. Parathyroid hormone (PTH) is assumed to have similar effects as PTHrP [8,9], as they share their N-terminus and receptor (PTHR1) [10,11]. Both PTH and PTHrP can enhance cartilage formation by stimulating the expression of SRY-box 9 [7] (SOX9, transcription factor required for chondrocyte differentiation and cartilage formation [12]) and by increasing cell proliferation through induction of cyclin D1 (CCND1) [13]. PTHrP/PTH have been demonstrated to stimulate chondrogenic differentiation of mesenchymal stromal cells (MSCs) and to prevent hypertrophic differentiation of MSCs [14–18] and growth plate chondrocytes [19,20] *in vitro*. Lastly, in rabbit osteochondral defects, intra-articular PTH administration stimulated tissue regeneration in vivo [21,22]. In contrast to osteochondral defect healing, ACI is based on chondrocyte implantation, either or not supplemented with MSCs [23]. Although expanding chondrocytes have stem cell-like properties [24], multilineage (especially osteogenic and adipogenic) differentiation efficacy is very low compared with MSCs, which may indicate that a chondroid precursor, but not a multipotent mesenchymal cell type is present in expanding chondrocytes [25].

As yet, it is unknown whether PTH may have similar effects on articular chondrocyte-mediated regeneration in terms of inhibition of hypertrophy and stimulation of regeneration. Therefore, in order to define whether PTH holds promise as an additive treatment strategy to current challenges faced by ACI, this study determined the effects of PTH on expanded human articular chondrocytes in an *in vitro* model of cartilage regeneration.

## Materials and methods

### Human chondrocytes

Healthy human femoral knee cartilage of three male and three female donors (mean age 68, range 47–83 years) was obtained post-mortem. Only macroscopically intact (Collins grade 0–1 [26]) cartilage was used (four samples grade 0, two samples grade 1). Collection of all patient material was done according to the Medical Ethical regulations of the University Medical Center Utrecht and according to the guideline ‘good use of redundant tissue for clinical research’ constructed by the Dutch Federation of Medical Research Societies on collection of redundant tissue for research (www.fedara.org). The material collected involved redundant material removed in the course of surgery and was used anonymously, without collection of patient data (article 7:467, Dutch civil code). This study does not meet the definition of human subjects research or require informed consent. Anonymous use of redundant tissue for research purposes is part of the standard treatment agreement with patients in our hospital [27].

### Chondrocyte isolation and expansion

Cartilage was digested overnight in Dulbecco’s modified Eagle Medium (DMEM; 42430, Invitrogen) containing 0.1% w/v collagenase type II (CLS2, Worthington), 1% v/v penicillin/streptomycin (P/S; 15140, Invitrogen). Isolated chondrocytes were washed in PBS and expanded at 5000 cells/cm^2^ in DMEM containing 1% P/S, 10% v/v fetal bovine serum (DE14-801F, Lonza), 10 ng/mL FGF (223-FB, R&D Systems). At 80% confluency, cells were trypsinized and passaged. Passage two cells were used for redifferentiation culture or snap-frozen for RNA analysis.

### Redifferentiation culture

Since more recent ACI versions are based on collagen carriers [28], the chondrocytes were cultured on collagen type II-coated membranes in a 24-wells transwell system as described previously [29]. Passage two chondrocytes were seeded on inserts (PICM01250, Millipore) with a hydrophilic poly-tetrafluoroethylene (PTFE) membrane at 1.6 x10^6^ cells/cm^2^ in DMEM with 2% w/v ITSx (51500, Invitrogen), 2% w/v ascorbic acid (A8960, Sigma-Aldrich), 2% w/v human serum albumin (HS-440, Seracare Life Sciences), 100 units/mL penicillin, 100 μg/mL streptomycin, and 10 ng/mL TGF-β_2_ (302-B2, R&D Systems). Before culture, the membranes had been coated with 0.125 mg/mL type II collagen (C9301, Sigma-Aldrich) in 0.1 M acetic acid. After thorough rinsing, efficient coating was verified by immunohistochemistry [30]. To mimic the different phases of maturation, PTH was supplied at different time points in two different concentrations: 0.1 or 1.0 μM PTH (based on Kafienah et al. (2007) [14]) was added every media change (three times a week) from days 0 (n = 4), 9 (n = 6) or 21 (n = 6) onwards, whereas the controls (n = 6) did not receive PTH. After 28 days, the tissues were fixed in 10% w/v neutral buffered formalin (for histological analysis) or snap-frozen and stored at -20 ºC (glycosaminoglycan (GAG) and DNA content analysis) or -80 ºC (for RNA analysis).

### Gene expression analysis

After separation of the neotissue from the transwell membranes with a scalpel (Swann-Morton), RLT with β-mercaptoethanol (M6250, Sigma) was added to the samples. Thereafter, samples were crushed and pestled with liquid nitrogen and 21G syringes until a homogeneous solution was obtained. RNA isolation was performed using the RNeasy Micro Kit (74004, Qiagen) according to the manufacturer’s protocol with DNAse treatment. RNA concentrations were measured using the NanoDrop ND-1000 spectrophotometer (NanoDrop Technologies). Sufficient RNA purity was assumed with 260/280nm ratio >1.9. Subsequently, 500 ng RNA was reverse transcribed using the iScript cDNA synthesis Kit (Bio-Rad). RT-qPCR was performed according to the manufacturer’s protocol (technical duplicates) using TaqMan Gene Expression Assays (Applied Biosystems). Primers for hypertrophic differentiation and (cartilaginous) extracellular matrix production were used: *COL1A1* (Hs00164004_m1), *COL2A1* (Hs00264051_m1), *COL10A1* (Hs00166657_m1), *RUNX2* (Hs00231692_m1, recognizes all three isoforms), and *MMP13* (Hs00942589_m1), all corrected for housekeeping gene *ACTB* (β-actin) (Hs99999903_m1). Primer specifications have been provided in S1 Table.

### Histological analysis

Histological evaluation was performed on 5 μm thick deparaffinized sections using Safranin O/Fast Green staining as described previously [31]. Cartilage quality by histology was assessed with the Bern Score [32], specifically designed for engineered cartilage, assessing intensity and uniformity of Safranin O staining, amount and organization of the extracellular matrix and cell morphology. The maximum score is 9, representing healthy hyaline cartilage.

### Immunohistochemistry

Immunohistochemical staining was performed for (cartilaginous) extracellular matrix production and (pre)hypertrophic differentiation: types I, II and X collagen, RUNX2, IHH and PTHR1. The sections were blocked with 0.3% H_2_O_2_ in PBS and 5% PBS/BSA (type I, II, and X collagen) or TBS/BSA 5% (RUNX2) and incubated overnight at 4 ºC with mouse monoclonal antibodies against type I collagen (20 μg/mL, CP-17, Calbiochem), type II collagen (0.4 μg/mL, ascites II-II6B3, Developmental Studies Hybridoma Bank), type X collagen (1:50 (actual concentration unknown), X53, Quartett) and RUNX2 (5 μg/mL, SC-101145, Santa Cruz) in 5% PBS/BSA. Subsequently, the samples were incubated with biotinylated sheep anti-mouse (1:200, RPN1001V, GE Healthcare) followed by peroxidase labeled streptavidin (1:500, IM0309 Beckman Coulter) (type I and X collagen) or goat anti mouse-HRP (1:100, P0447, DAKO) (type II collagen, RUNX2) for 1 hour in 5% PBS/BSA. Sections were counterstained with hematoxylin. Juvenile growth plate cartilage was used as a positive control. Sections were incubated with isotype antibodies as negative controls. Negative control sections did not show staining, whereas positive control sections showed specific staining (data not shown).

To determine IHH (sc-1196, Santa cruz, 4 μg/mL) and PTHR1 (sc-12777, Santa Cruz, 10 μg/mL) expression, sections were immunohistochemically stained using the ImmunoCruz goat LSAB Staining System (sc-2053, Santa Cruz) with citrate buffer (10 mM, pH 6) antigen retrieval (30 minutes, 70°C) [19].

### GAG and DNA analysis

GAG and DNA content of the tissue constructs were digested in 2% papain (P3125, Sigma-Aldrich) in 50 mM phosphate buffer, 2 mM N-acetylcysteine, and 2 mM Na_2_-EDTA (pH 6.5) at 65°C for 2 hours. GAGs were precipitated from tissue digests and culture medium (each medium change) using Alcian blue dye (Alcian blue 8GX, 05500, Sigma-Aldrich) saturated in 0.1 M sodium acetate buffer containing 0.3 M MgCl_2_ (pH 6.2) for 30 min at 37°C [33]. Absorbance was quantified photospectrometrically (520 nm) with chondroitin sulphate (C4384, Sigma) as reference. DNA was quantified by Hoechst 33258 (94403, Sigma) staining [34] followed by fluorescence measurement (Cytofluor, Bio-Rad) with calf thymus DNA (D4764, Sigma) as reference.

### Statistics

Statistical analyses were performed using IBM SPSS 20 for Windows (IBM SPSS Inc.). Data were tested for normality using the Kolmogorov–Smirnov test and for homogeneity of variances using the Levene’s equality of variances test. Subsequently, mixed model analysis (random effect: donor, fixed effect: PTH concentration and moment of initiating treatment) was performed. After performing a Bonferroni *posthoc* test for multiple comparisons, p<0.05 was considered statistically significant.

## Results

### PCR

**Fig 1:**
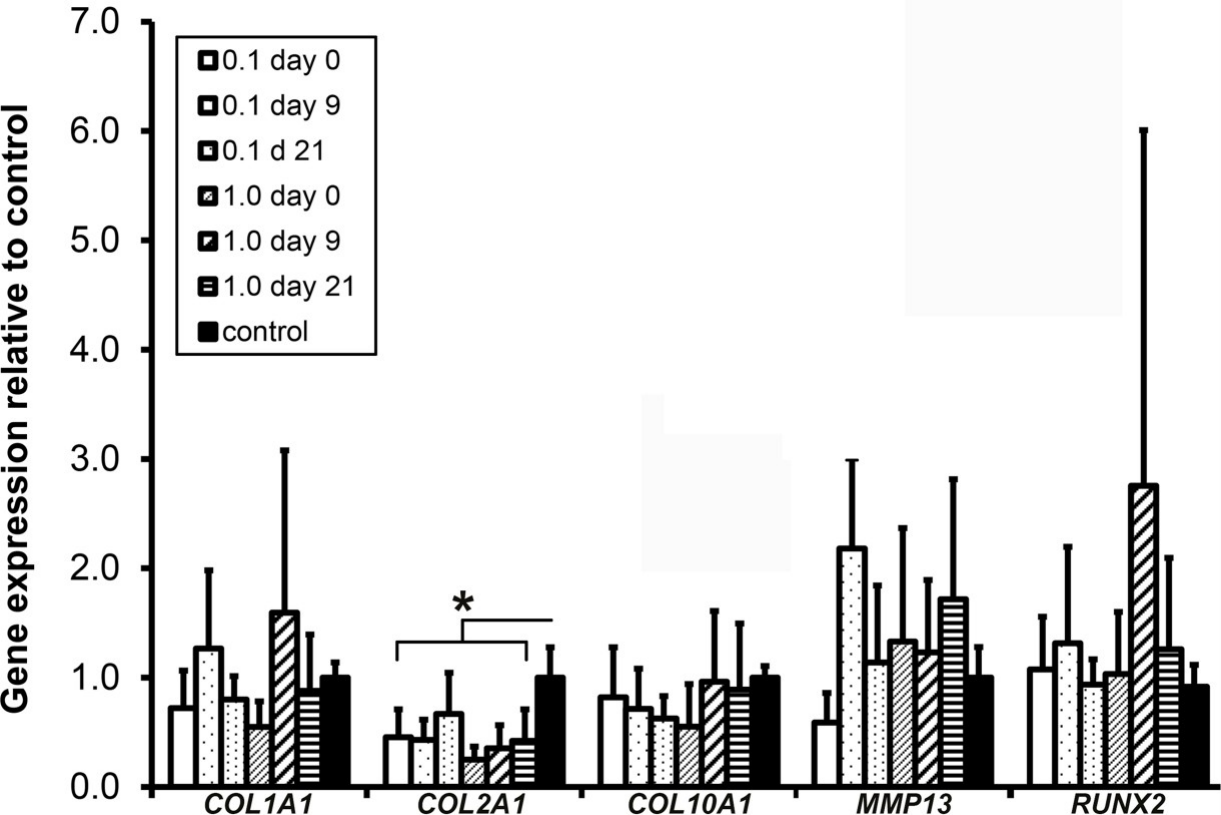
Gene expression of type I, II and X collagen (*COL1A1*, *COL2A1*, *COL10A1*), matrix metallopeptidase 13 (*MMP13*) and runt related transcription factor 2 (*RUNX2*) by human chondrocytes cultured for 28 days in the presence of 0.1 or 1.0 μM PTH from day 0, 9 or 21 onwards. Controls did not receive PTH. 0.1 or 1 μM PTH was added from day 0, 9 or 21 onwards. *: p = 0.042. Graphs show mean ± 95% C-I. n = 6.

In all PTH-treated neotissues, *COL2A1* mRNA expression was decreased compared with controls (p = 0.042, Fig 1). No effect of PTH was observed on *COL1A1*, *COL10A1*, *MMP13*, or *RUNX2* expression.

### Bern score and immunohistochemistry

**Fig 2:**
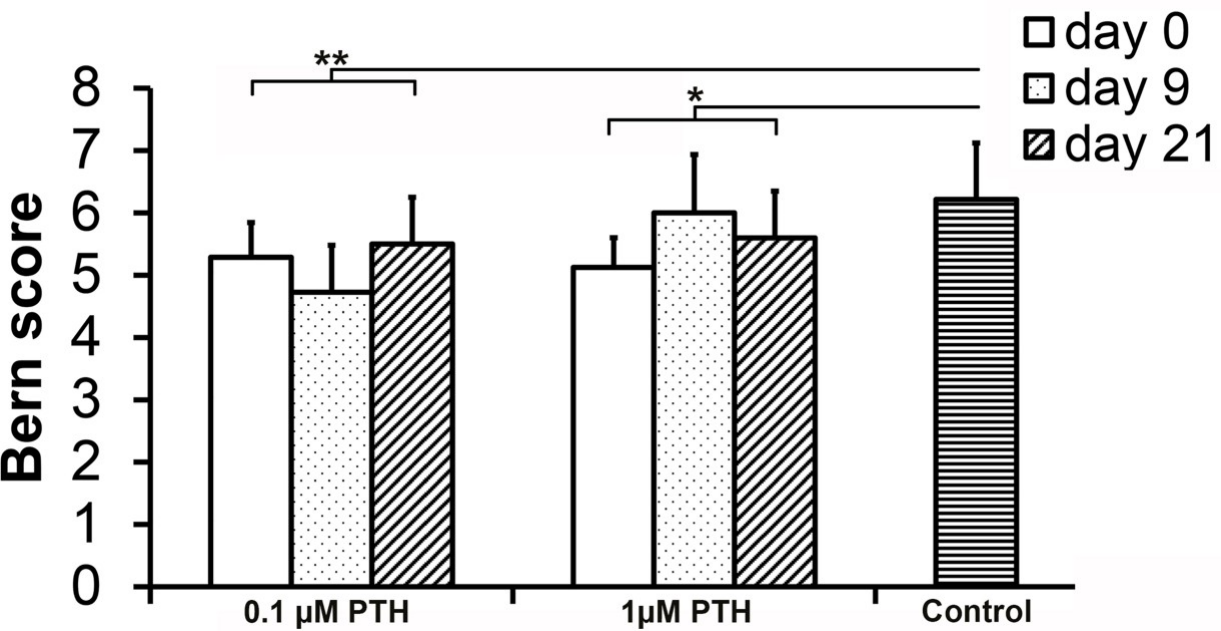
Bern score of human chondrocytes cultured for 28 days in the presence of 0.1 or 1.0 μM PTH from day 0, 9 or 21 onwards. Controls did not receive PTH. 0.1 μM and 1 μM PTH addition resulted in lower Bern scores compared with controls (*: p = 0.006, **: p = 0.022, respectively). No differences were observed between PTH concentrations or moment of supplementation. Graphs show mean ± 95% C-I. n = 6.

**Fig 3:**
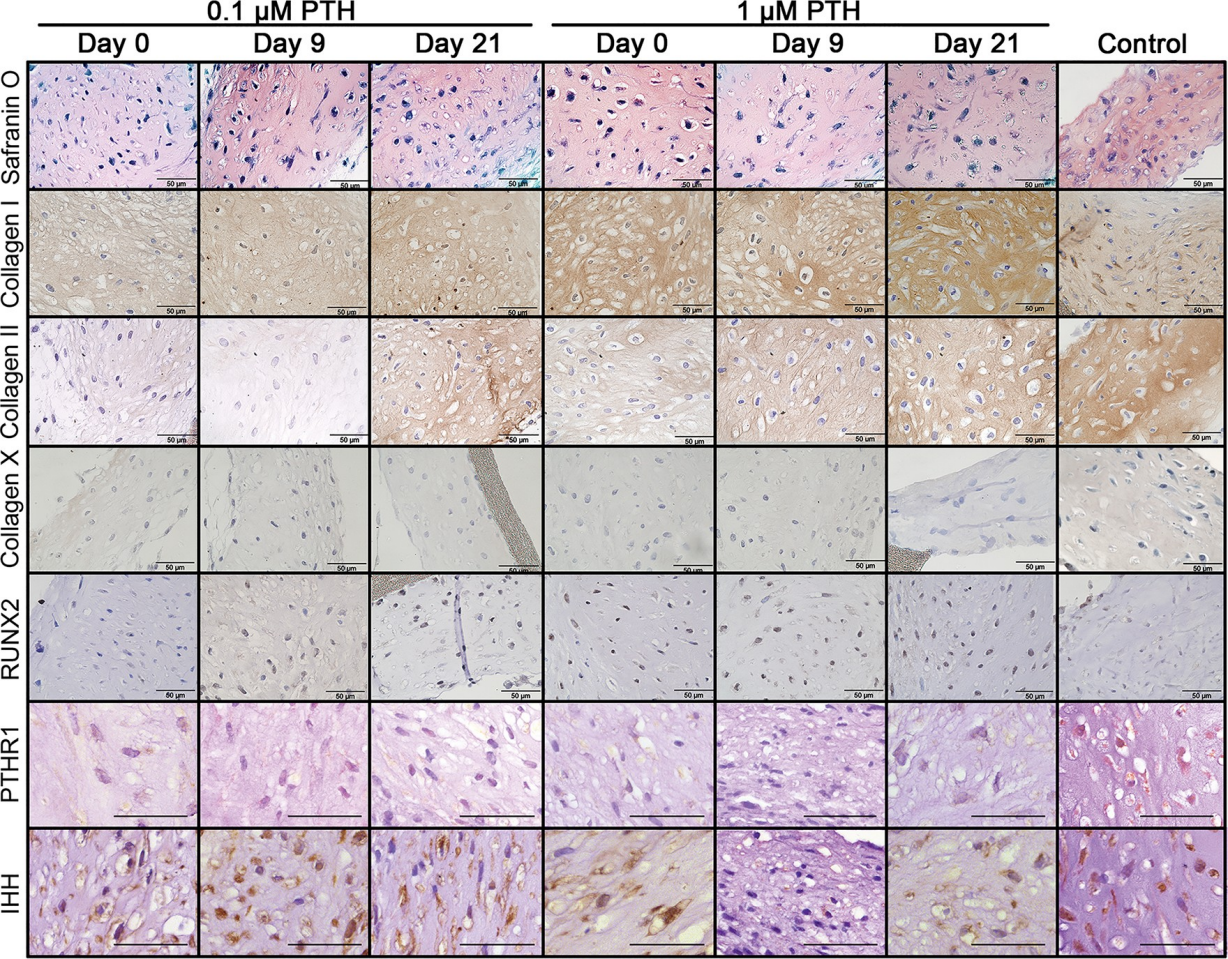
Histological sections of human chondrocytes cultured for 28 days in the presence of 0.1 or 1.0 μM PTH from day 0, 9 or 21 onwards. Controls did not receive PTH. All scale bar represent 50 μm. n = 6.

Both 0.1 μM PTH (p = 0.006) and 1 μM PTH (p = 0.022) treatment resulted in lower Bern scores compared with controls, regardless the moment of initiating treatment (Fig 2). Safranin O staining intensity was not different between conditions. Type I collagen deposition appeared higher in (mainly 1.0 μM) PTH-treated cultures compared with controls (Fig 3). Furthermore, type II collagen deposition was slightly lower in all PTH-treated cultures compared with controls, most profoundly in 0.1 μM PTH-treated chondrocytes. No type X collagen and RUNX2 deposition was observed in any condition. IHH was abundantly present in all donors and conditions, but was not differentially expressed. PTHR1 expression appeared absent after 0.1 and 1.0 μM PTH treatment (irrespective of initiation of treatment), whereas control chondrocytes showed weak membranous PTHR1 expression.

### GAG and DNA content, GAG production and release

**Fig 4:**
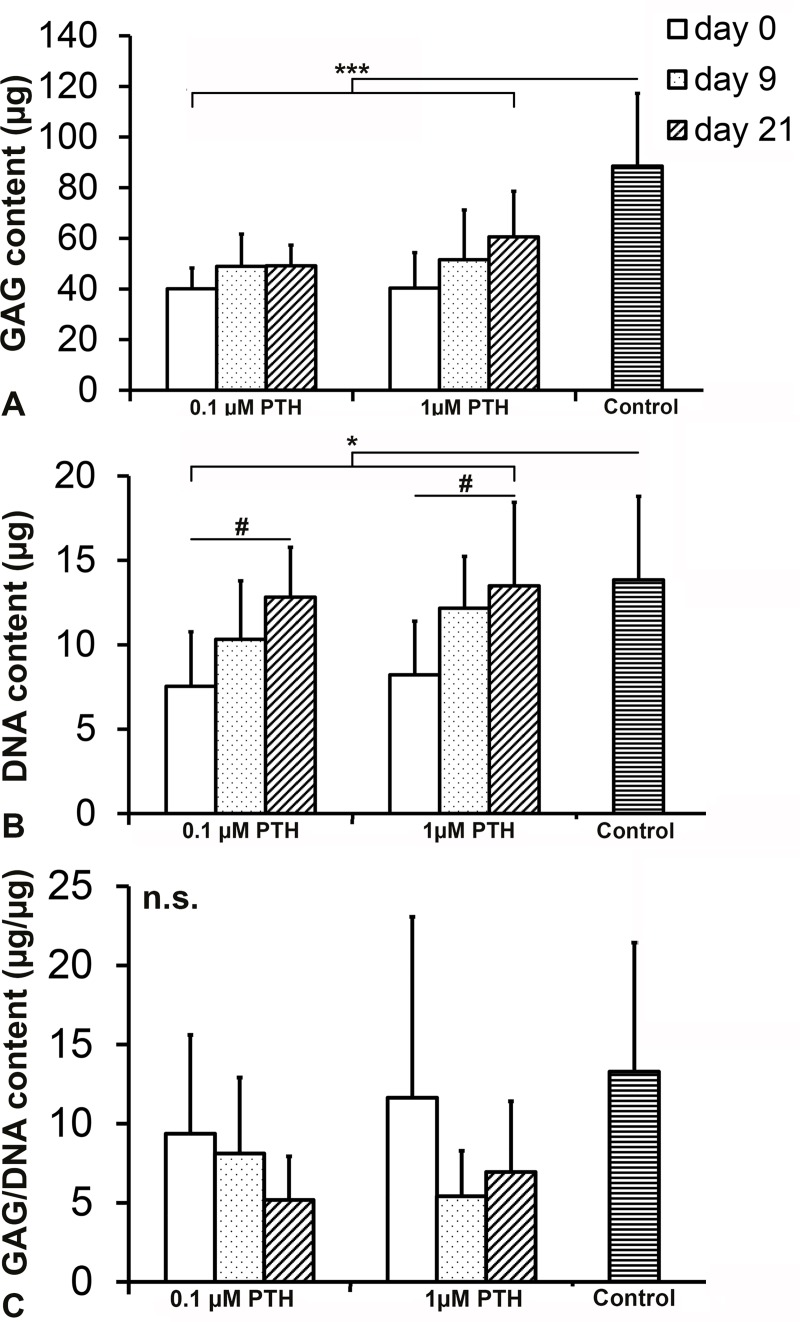
GAG, DNA and GAG/DNA content of human chondrocytes cultured for 28 days in the presence of 0.1 or 1.0 μM PTH from day 0, 9 or 21 onwards. Controls did not receive PTH. (a) PTH treatment resulted in a lower GAG content (p = 0.001). (b) DNA content was decreased by addition of 0.1 and 1.0 μM PTH (*: p = 0.030). A time-dependent increase in DNA content was observed in the presence of PTH (#: p = 0.027), with no difference between 0.1 and 1.0 μM. (c) GAG/DNA content did not differ between conditions. n.s.: not significant. Graphs show mean ± 95% C-I. n = 6.

**Fig 5:**
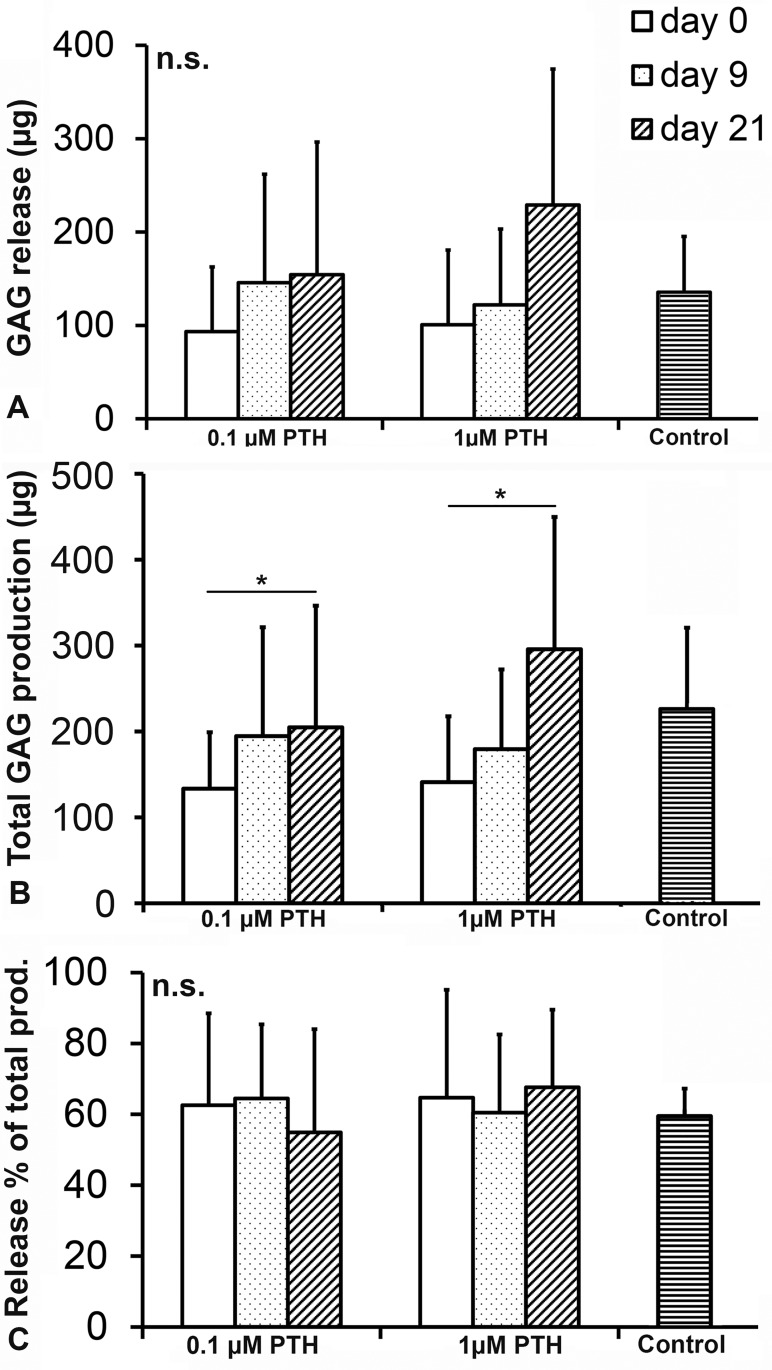
Total GAG production, total GAG release and GAG release as percentage of total production of human chondrocytes cultured for 28 days in the presence of 0.1 or 1.0 μM PTH from day 0, 9 or 21 onwards. Controls did not receive PTH. Total GAG production was dependent on moment of PTH supplementation (*: p = 0.042), but not on PTH concentration. n.s.: not significant. Graphs show mean ± 95% C-I. n = 6.

PTH treatment decreased the constructs’ GAG content compared with controls, regardless of the concentration or initiation of treatment (p = 0.001, Fig 4A). Also, DNA content of the constructs was decreased by the addition of 0.1 and 1.0 μM PTH (p = 0.030, Fig 4B). For both PTH concentrations, DNA content was inversely related to the duration of PTH exposure (p = 0.027). The GAG content corrected for DNA content (indirect measure of GAG deposition per cell) was not influenced by the addition of PTH (p = 0.482, Fig 4C). GAG release and total GAG production (sum of total amount of GAGs released into the medium and GAG content of the regenerated tissue) were also not affected by PTH treatment (p = 0.586, Fig 5A and 5B). Relative GAG release (release as percentage of total production) did not differ between the different PTH concentrations or controls (p = 0.656, Fig 5C).

## Discussion

Although ACI is effective at treating medium-sized cartilage defects [1], the newly formed cartilage can be of reduced (e.g. hypertrophic) quality [3]. PTH and PTHrP have the ability to inhibit hypertrophic differentiation of MSCs [14–18] and growth plate chondrocytes [19,20]. Therefore, the first aim of this study was to determine whether PTH could also inhibit hypertrophic differentiation in healthy human chondrocytes, the cell source used for ACI.

Although both PTHR1 and IHH are considered prehypertrophic differentiation markers [35], only PTHR1 expression was inhibited by PTH supplementation, in line with previous work on growth plate chondrocytes [9,19]. Since IHH, RUNX2 and type X collagen (hypertrophic differentiation marker [35]) expressions were not inhibited by PTH, the downregulation of PTHR1 presumably reflects a negative feedback mechanism to prevent overstimulation [19], rather than an inhibition of (pre)hypertrophic differentiation. The absence of a PTH-mediated effect on hypertrophic differentiation is in contrast with previously observed effects on hypertrophic articular [36] and growth plate [19] chondrocytes. Although diminished responsivity to PTH has been reported for chondrocytes [14,20], this is unlikely the cause of the lack of effect on hypertrophic differentiation, since the DNA content and associated extracellular matrix composition were affected by PTH. Presumably, the chondrocytes reached at least a prehypertrophic state in the current study, since PTHR1 and IHH were expressed in controls. However, since no clear features of hypertrophic differentiation (e.g. hypertrophic cellular phenotype, abundant type X collagen deposition) were observed, other factors may have been required for its induction, such as present in hypertrophic induction medium [37], e.g. phosphate [38]. However, it remains speculative to what extent the factors in hypertrophic media induce hypertrophic differentiation in vivo [39], as until now these have remained elusive.

Since PTHrP stimulates growth plate chondrocyte proliferation [35], the inhibitory effect of PTH on the tissue constructs’ DNA content was rather surprising. However, the direction of the PTH-mediated effects appears to be dependent on dose [40,41] and maturation phase [42]. The latter is stressed by the fact that PTH was able to increase the mitogenic response of growth plate chondrocytes, but not of healthy articular chondrocytes [43]. The question arises which difference between growth plate and articular chondrocytes may explain this differential response to PTH. While at a gene expression level there are similarities in the spatial architecture of growth plate and articular cartilage [44], their embryonic origin is distinctly different. Articular chondrocytes are embryonically derived from the interzone cells forming both the synovial lining and the articular cartilage of the joint [45] and in postnatal stages can be distinguished from growth plate chondrocytes by lubricin expression [46]. Possibly, the signaling mechanisms are also different between articular and growth plate chondrocytes. Lastly, the inhibitory effect of PTH on the DNA content could have been caused by signaling via a different receptor. For example, binding of the PTH family member TIP39 to PTHR2 has been shown to inhibit articular chondrocyte proliferation [47]. Future studies should further determine the age- and possibly OA-dependent expression and effects of PTHR2 versus PTHR1 signaling in articular chondrocytes.

0.1 μM PTH treatment has been shown to stimulate collagen type II deposition in hypertrophic articular chondrocytes [36]. Therefore, the second aim of this study was to determine whether PTH could also enhance cartilaginous (GAG- and type II collagen-rich) extracellular matrix deposition by healthy human articular chondrocytes. Mainly due to the decrease in cell number, GAG and type II collagen deposition were inhibited by 0.1 and 1 μM PTH in the current study. The latter is in line with results obtained with rat articular chondrocytes [11] and chicken sterna [48]. Possibly, lowering the PTH concentration to below 0.1 μM could have prevented the observed inhibition of type II collagen deposition [49], since PTH is known to exert opposite effects at low versus high concentrations in this respect [40,41]. While *COL1A1* mRNA expression was not significantly different between conditions, 1.0 μM PTH-treated cultures showed increased type I collagen deposition. It is a common drawback of relative gene expression studies that mRNA expression is not always accompanied by similar protein expression patterns [50]. RT-qPCR demonstrates mRNA expression at that specific (snapshot) moment, whereas collagen type I IHC results show a cumulative effect over time. Presumably, *COL1A1* mRNA expression was already normalized after an initial increase by PTH treatment or differed from protein expression due to posttranslational processes (e.g. epigenetics).

When considering the clinical application of PTH for the repair of cartilage defects, intermittent PTH administration seems favorable to continuous administration: it enhanced chondrogenesis and suppressed hypertrophy of human MSCs compared with continuous administration [17,18], improved the quality of regenerated rabbit cartilage [22] and prevented osteoporosis in postmenopausal women [51]. In the current study, PTH was administered continuously instead of intermittently, possibly explaining the absence of a beneficial effect. However, local sustained delivery resulted in similar protective effects against GAG loss and type X collagen deposition in a rat OA model as obtained with intermittent injection of PTH [52], suggesting that local delivery may overcome the requirement for intermittent exposure.

The inhibition of cartilage regeneration observed in our study would argue against the use of PTH in cartilage repair procedures based on ACI. Although PTH did not exert any positive effects on healthy human articular chondrocytes, it could still be beneficial for cartilage repair based on MSCs alone [14–18] (e.g. microfractures) or procedures in which an overwhelming concentration of MSCs is combined with chondrocytes, for example during a one-stage cartilage repair procedure. Previous work indeed indicated that PTH-induced rat bone marrow-derived MSCs complexed with fibrin glue effectively promoted repair of a rabbit articular cartilage injury [53]. Stem cells are usually derived from fat or bone marrow [54], but also other cell sources have been proposed, such as human dental pulp stem cells [55] or follicle cells enveloping the dental germ [56]. However, care should be taken not to interfere with endogenous repair by for example the cartilage progenitors or interzone-derived MSCs from the synovial lining, which are known to contribute to the repair of cartilage defects [45]. Lastly, PTH treatment could potentially be combined with chondroitin-based scaffolds, since biotechnological chondroitin has been shown to preserve the chondrocytic phenotype and reduce inflammation [57].

## Conclusions

Continuous PTH treatment inhibited regeneration of healthy human articular chondrocytes, while hypertrophic differentiation was not influenced, indicating that articular chondrocytes respond differently to PTH compared with MSCs. Therefore, PTH may be more suitable for cartilage repair based on MSCs than on articular chondrocytes.

## Supporting information

S1 Table Primer specifications. RT-qPCR was performed according to the manufacturer’s protocol (technical duplicates) using TaqMan Gene Expression Assays (Applied Biosystems). Thermofisher does not provide primer sequences.
(DOCX)
