# Supplementary material for: PTH decreases in vitro human cartilage regeneration without affecting hypertrophic differentiation
Source: PLoS One. 2019 Apr 4;14(4):e0213483. doi: 10.1371/journal.pone.0213483 (PMC6449021; doi:10.1371/journal.pone.0213483)
Supplement: S1 Table — RT-qPCR was performed according to the manufacturer’s protocol (technical duplicates) using TaqMan Gene Expression Assays (Applied Biosystems). Thermofisher does not provide primer sequences. (DOCX) [file pone.0213483.s001.docx]

Supplementary file 1: Primer specifications.

| Target/reference gene | Assay ID | Amplicon length (bp) | Exon boundary | Assay location (bp) | Annealing temperature (°C) |
| --- | --- | --- | --- | --- | --- |
| *COL1A1* | Hs00164004_m1 | 66 | 1-2 | 230 | 57 |
| *COL2A1* | Hs00264051_m1 | 124 | 7-8 | 712 | 57 |
| *COL10A1* | Hs00166657_m1 | 76 | 2-3 | 250 | 57 |
| *RUN*X2 | Hs00231692_m1, recognizes all three isoforms | 116 | 5-6 | 900 | 57 |
| *MMP13* | Hs00942589_m1 | 117 | 7-8 | 1086 | 57 |
| *ACTB* | Hs99999903_m1 | 171 | 1 | 53 | 57 |

RT-qPCR was performed according to the manufacturer’s protocol (technical duplicates) using TaqMan® Gene Expression Assays (Applied Biosystems). Thermofisher does not provide primer sequences.
